# *Shewanella oneidensis* MR-1 as a bacterial platform for electro-biotechnology

**DOI:** 10.1042/EBC20200178

**Published:** 2021-07-26

**Authors:** Sota Ikeda, Yuki Takamatsu, Miyu Tsuchiya, Keigo Suga, Yugo Tanaka, Atsushi Kouzuma, Kazuya Watanabe

**Affiliations:** School of Life Sciences, Tokyo University of Pharmacy and Life Sciences, 1432-1 Horinouchi, Hachioji 192-0392, Tokyo, Japan

**Keywords:** bioelectrochemistry, bioenergetics, biofilm, bioproduction, fermentation

## Abstract

The genus *Shewanella* comprises over 70 species of heterotrophic bacteria with versatile respiratory capacities. Some of these bacteria are known to be pathogens of fishes and animals, while many are non-pathogens considered to play important roles in the global carbon cycle. A representative strain is *Shewanella oneidensis* MR-1 that has been intensively studied for its ability to respire diverse electron acceptors, such as oxygen, nitrate, sulfur compounds, metals, and organics. In addition, studies have been focused on its ability as an electrochemically active bacterium that is capable of discharging electrons to and receiving electrons from electrodes in bioelectrochemical systems (BESs) for balancing intracellular redox states. This ability is expected to be applied to electro-fermentation (EF) for producing value-added chemicals that conventional fermentation technologies are difficult to produce efficiently. Researchers are also attempting to utilize its electrochemical ability for controlling gene expression, for which electro-genetics (EG) has been coined. Here we review fundamental knowledge on this bacterium and discuss future directions of studies on its applications to electro-biotechnology (EB).

## Introduction

The genus *Shewanella* comprises over 70 species and includes bacterial strains isolated from diverse ecosystems, including freshwater sediment, deep-sea sediment, oil brine, fish bodies, and spoiled foods [1,2]. These are Gram-negative, facultatively anaerobic, heterotrophic bacteria, some of which are known to be pathogens of fishes and animals [3,4]. Most of them are easily cultivable in nutrient-rich media, and enrichment and isolation procedures for these bacteria are described elsewhere [1]. To date, genomes of more than 200 strains in the genus *Shewanella* have been sequenced, and comparative genomic analyses have suggested that these bacteria share common genetic features [5].

A distinctive feature of bacteria affiliated with the genus *Shewanella* is versatile respiratory capacity [1,2]; namely, they are capable of respiring various inorganic and organic compounds, including, oxidized metals, such as Fe(III) and Mn(III, IV), metal oxides, such as magnetite and hematite, nitrate, elemental sulfur, sulfite, thiosulfate, arsenate, fumarate, trimethylamine-N-oxide, and dimethyl sulfoxide. This capacity is supported by the presence of various terminal reductases and overlapping electron-transport pathways, facilitating them to survive in redox-stratified environments, where available electron acceptors repeatedly change in response to fluctuations in environmental conditions [5]. In natural habitats, these bacteria tend to grow by decomposing organic compounds produced by fermentative bacteria, such as lactate and amino acids, and exhausting carbon dioxides and/or acetate with the expense of available electron acceptor(s) [1,2]. Understanding of the physiology of *Shewanella* is therefore important for gaining ecological insights into organic matter decomposition in natural ecosystems.

Some electron acceptors that *Shewanella* is able to utilize, such as metal oxides, are solid and insoluble, and these bacteria have evolved specialized electron-transport pathways, termed ‘extracellular electron-transport (EET) pathways’ for transferring electrons extracellularly to solid electron acceptors [5,6]. Using the EET pathways, they are also able to exchange electrons with electrodes in bioelectrochemical systems (BESs) [7] and are therefore termed ‘electrochemically active bacteria (EAB)’ [8]. To date, BESs have been examined for their application to a variety of biotechnology processes, including, microbial fuel cells (MFCs), microbial electrolysis cells (MECs), and microbial electrosynthesis (MES) [9]. Among these, MFCs are expected to be applied to power generation from biomass wastes [10], while MECs are designed to generate hydrogen gas, CO_2_-free clean energy, at high efficiencies [11]. MES is also attractive in its sustainable production of organic compounds from CO_2_ [12]. Since EAB play pivotal roles in these BESs, researchers focus their efforts to understand the physiology and genetics of EAB for better utilizing these bacteria in BESs.

In addition, the capacity of EAB to exchange electrons with electrodes is also applicable to the control of intracellular redox states using electrodes. Different from conventional fermentation processes, in which the total redox equivalent must be balanced between substrates and products, bioprocesses using EAB are considered to be able to produce either oxidized or reduced products at high yields using electrodes as electron scavengers or suppliers, respectively, and such a technology has been termed ‘electro-fermentation (EF)’ [13,14]. In addition, recent studies have proposed ‘electro-genetics (EG)’, in which gene expression in EAB is regulated by intracellular redox-sensing systems whose function is controllable using electrodes in BESs [15]. These emerging technologies would further increase the value of EAB, while further efforts are necessary for bringing these technologies close to practical applications. In such work, in terms of annotated genome sequence [16], genetic accessibility [6], and extensive knowledge on its molecular mechanisms [15,17], *Shewanella oneidensis* MR-1 serves as a model EAB.

In this article, we summarize fundamental knowledge on *S. oneidensis* MR-1 and discuss its potential applications to biotechnology. Since efficiencies of biotechnology processes carried out in BESs (electro-biotechnology, EB) are determined by metabolic and electrochemical properties of EAB as well as their interaction with electrodes as biofilms, we review knowledge on these features of strain MR-1 and suggest future directions of studies for developing efficient EB processes. We will conclude that this bacterium serves as a bacterial platform for developing EB with broad applications.

## Metabolic characteristics of *S. oneidensis* MR-1 in relation to biotechnology

**Figure 1:**
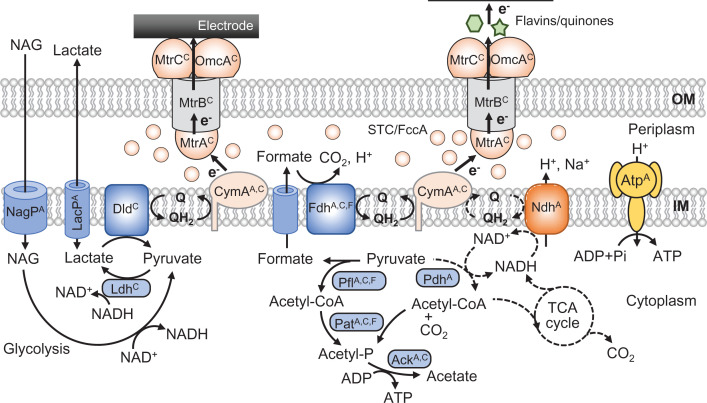
Catabolic and electron-transfer pathways in *S. oneidensis* MR-1 for anodic current generation Broken lines indicate steps down-regulated in the presence of low-potential electrodes. Catabolic enzymes and transporters are indicated in blue, cytochromes in the EET pathway in pale orange, NADH dehydrogenase in orange, and ATP synthase in yellow. Superscript A indicates enzymes whose gene expression is regulated by the Arc system, superscript C indicates enzymes whose gene expression is regulated by the cAMP/CRP system, while superscript F indicates enzymes whose gene expression is regulated by the Fnr system. Abbreviations: Ack, acetate kinase; Atp, ATP synthetase; cAMP/CRP, cyclic AMP/cAMP-receptor protein; Dld, d-lactate dehydrogenase; Fdh, formate dehydrogenase; LacP, lactate permease; Ldh, lactate dehydrogenase (fermentative); NagP, NAG permease; Ndh, NADH dehydrogenase; IM, inner membrane; OM, outer membrane; Pat, phosphate acetyltransferase; Pdh, pyruvate dehydrogenase; Pfl, pyruvate formate lyase; Q, oxidized quinone; QH_2_, reduced quinone.

Genomic and genetic studies have assembled catabolic and electron-transport pathways in *S. oneidensis* MR-1 (Figure 1), and enzymes involved in these pathways are detailed elsewhere [17,18]. This bacterium prefers to utilize low molecular weight organic compounds, such as lactate, pyruvate and amino acids, as carbon and energy sources, while its ability to metabolize sugars is limited, except for the ability to utilize N-acetylglucosamine (NAG), an abundant sugar in the marine environment [19]. An interesting feature in sugar catabolism in MR-1 is that this bacterium utilizes d-lactate as a temporal electron sink in the process to produce acetate as the catabolic end product under electron acceptor-limited conditions [20] (Figure 1). MR-1 is however unable to grow on glucose, an abundantly present sugar in the natural environment and a widely used feedstock in biotechnology processes [17]. Genomic analyses have suggested that the inability in glucose utilization is attributable to the deficiencies in the uptake and initial phosphorylation steps [17]. This idea has been confirmed in a study that shows that the introduction of genes encoding sugar permease and hexose kinase in *Escherichia coli* facilitates MR-1 to grow on glucose in the presence of electron acceptors, e.g. oxygen and fumarate [21]. In addition, it has also been shown that this engineered strain generates anodic current in glucose-supplemented BES via a working electrode (WE) poised at +0.6 V (vs. a standard hydrogen electrode, SHE) and produces lactate as an intermediate metabolite and acetate as the end product [21]. This engineered strain however does not grow on glucose under fermentative conditions (in the absence of electron acceptors), even though it has all enzymes necessary for completing the fermentative pathway from glucose to lactate [18]. Currently, it is not clear why the engineered strain cannot grow on glucose fermentatively, while understanding the reasons and development of methods to overcome this deficiency would be very important to utilize MR-1 and its derivatives in EF for producing value-added chemicals from biomass feedstocks.

It is also important to note that the expression of many genes for catabolic and electron-transport pathways in *S. oneidensis* MR-1 is regulated by global regulatory systems; among these, a cyclic AMP (cAMP)/cAMP-receptor protein (CRP) system and an aerobic respiration control (Arc) system are considered to play central roles [18]. For instance, the expression of genes for the EET pathway, including *mtrABC* and *omcA*, is regulated by the cAMP/CRP system [22]. It has also been found that this system regulates transcription of genes for lactate permease and respiratory d-lactate dehydrogenase [23]. MR-1 also has an Arc global regulatory system comprising sensor histidine kinase (ArcS), phosphotransfer protein (HptA), and response regulator (ArcA) [24,25], and studies have shown that this system controls transcription of many genes for energy metabolism in MR-1, including genes for NADH dehydrogenase (*nuo*), pyruvate dehydrogenase (*aceE*), and ATP synthetase (*atp*) [26,27]. In addition to the cAMP/CRP and Arc systems, other global regulators, including homologs of ferric uptake regulator (Fur) and fumarate nitrate reduction regulator (Fnr) in *E. coli*, are also involved in the transcriptional regulation of catabolic genes [28,29]. Furthermore, some catabolic genes are regulated by multiple global regulatory systems [30], suggesting the presence of complex regulatory cascades in MR-1. Since such genes encode pivotal enzymes in the bioconversion and electron-transfer pathways, deep understanding of the regulatory cascades is necessary for developing efficient EB processes.

Among global regulators, the Arc system would be of particular focus in bioelectrochemistry, since it senses redox states of membrane quinones for regulating the expression of catabolic genes [24]. Furthermore, a recent work has found that MR-1 changes catabolic pathways depending on electrode potentials in BESs, in which the Arc system is involved [27]. That work has shown that the *nuo* and *aceE* genes are up-regulated in the presence of high-potential electrodes (e.g. +0.6 V vs. SHE), resulting in the activation of the NADH-dependent pathway at high potentials (see Figure 1). Since the NADH-dependent pathway translocates more protons per electron than the formate-dependent pathway, MR-1 is able to generate large proton-motive force in the presence of high-potential electrodes [27]. It is likely that this would be a mechanism in MR-1 and other bacteria for conserving an appropriate amount of biological energy (ATP synthesis) depending on an electromotive force of a catabolic reaction. Although this mechanism is of value for MR-1 to efficiently conserve energy in nutrient-limited natural environments, low-level expression of the NADH-dependent pathway at low potentials would be problematic in cathodic EF for producing reduced chemicals. However, since the MR-1 genome encodes 4 NADH dehydrogenases (Ndhs, including one proton-pump Ndh [Nuo], two sodium-pump Ndhs, and one non-pump Ndh) [18], studies should also be conducted to identify Ndh(s) that function at low-potentials.

## Electrochemical characteristics of MR-1 in relation to biotechnology

**Figure 2:**
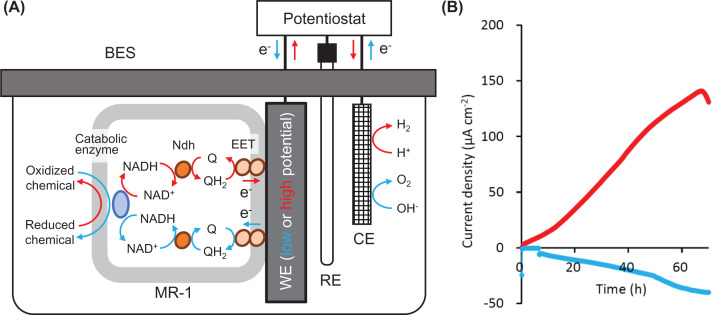
Anodic and cathodic current generation by *S. oneidensis* MR-1 in BESs (**A**) Three-electrode BES designed for EF, and possible major reactions in the presence of a WE poised at a low (blue) or high (red) potential. RE is a reference electrode, while CE is a counter electrode. (**B**) Typical chronoamperometric records of anodic (red) and cathodic (blue) currents from BESs inoculated with MR-1. Anodic current was generated with lactate as an electron donor in the presence of WE at +0.6 V (vs. SHE), while cathodic current was generated with fumarate as an electron acceptor in the presence of WE at −0.4 V. Current density was calculated based on a projected area of WE. Refer to [27] for experimental details.

Understanding of electrochemical characteristics is essential for utilizing and controlling EAB in BESs applied to biotechnology, since cell voltage is directly linked to power output from MFC, and charged voltage is related to electricity expense in MEC. Electrochemical characteristics of EAB have been analyzed using three-electrode BESs connected to potentiostats [31] (Figure 2A). In BESs, EAB grow in association with a WE, and electrochemical activities of EAB are measured as electric currents via WEs, whose direction and amount are determined by various factors, including WE potentials, chemical compounds in electrolytes, the quantity of microbial cells connected to WE, and microbial metabolic potentials [32]. For instance, in the presence of reduced chemicals (electron donors for EAB) in an electrolyte and a high-potential WE, anodic current (electron transfer from EAB cells to WE) is generated, whereas, in the presence of oxidized chemicals (electron acceptors for EAB) and a low-potential WE, cathodic current (electron transfer from WE to EAB cells) is generated (Figure 2). Chronoamperometry is a method for growing EAB and analyzing their electrochemical activities (measured as anodic or cathodic current) at a constant WE potential [33] (Figure 2B), which can also be used for electricity-assisted bioconversion of chemicals in EF. A study has conducted chronoamperometric measurements of anodic currents from strain MR-1 at different WE potentials and found that growth yields (measured as proteins synthesized on WE per electrons transferred as anodic current) are high in the presence of high-potential WEs [27].

In other studies, cyclic voltammetry (CV) has been used for examining electrochemical characteristics of whole MR-1 cells [34–37] and its electron-transfer components [38]. In CV, WE potential is repeatedly sweeped between two values for measuring current under potential-changing conditions. Whole-cell CV analyses of strain MR-1 have shown that soluble flavins either secreted by MR-1 or artificially added to an electrolyte accelerate anodic and cathodic currents [34]. In addition, the study has also found that, in the absence of flavins, electron transfer between WE and MR-1 cells occurs in a broad potential window centered at 0 V (vs. SHE), whereas, in the presence of flavins, electron transfer occurs at lower potentials (centered at −0.2 V) [34]. Another study used CV for characterizing electrochemical features of EET components in MR-1 (OmcA, MtrC, MtrA, STC and CymA, see Figure 1), showing that, although midpoint potentials of outer-membrane components (OmcA, MtrC and MtrA) are higher than those of periplasmic and inner-membrane components (STC and CymA), their potential windows are wide and largely overlap with each other [38]. This result suggests that, despite that the EET pathway in MR-1 is preferable for transporting electrons from the inside of microbial cells to the outside, this pathway can be used for electron transfers in both directions (those for anodic and cathodic currents, Figure 2B). This feature of the EET pathway would reflect the ecology of this bacterium; namely, it thrives at the bottom of lake Oneida, an organics-rich habitat with solid electron acceptors (iron and manganese crusts at the lake beds) [39]. In addition, the above-mentioned features support the idea that the EET pathway of MR-1 is applicable to EF for producing either oxidized or reduced products. It is however likely that its electron-transfer rate may have to be increased for developing efficient BES technologies, in particular, those for bioproduction of reduced chemicals in cathodic EF with the intake of electrons from a low-potential WE (see Figure 2B). As described below, understanding of mechanisms for biofilm formation on WE would also be necessary for this.

## Biofilm formation for electrochemical interactions with electrodes

**Figure 3:**
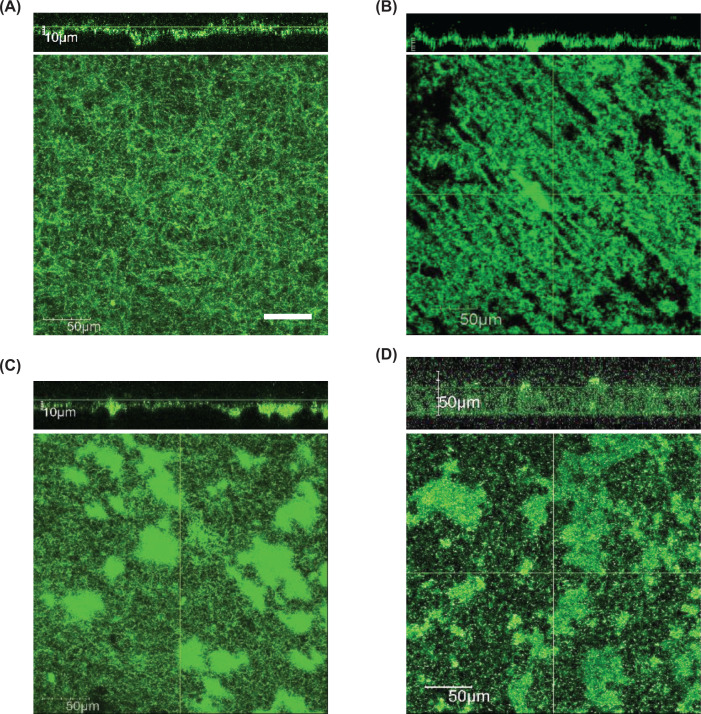
Biofilms formed by *S. oneidensis* MR-1 on graphite electrodes in EFCs under different conditions (**A**) Mature biofilm formed under an anaerobic, anodic current-generating and electrolyte-flow condition in the presence of WE poised at +0.4 V (vs. SHE). (**B**) Mature biofilm formed under an anaerobic, anodic current-generating and electrolyte non-flow (static) condition in the presence of WE poised at +0.4 V. (**C**) Mature biofilm formed under an anaerobic, anodic current-generating and electrolyte-flow condition in the presence of WE poised at 0 V. (**D**) Mature biofilm formed under an aerobic, open-circuit and electrolyte-flow condition. For each condition, x-z (top) and x-y (bottom) images are shown. A scale bar in panel (A) (50 μm) applies to all images. Refer to [42] for experimental details. Copyright © American Society for Microbiology.

Studies have shown that EAB cells adhere to electrodes and form biofilms for efficient electrochemical interaction with electrodes [40]. It is therefore important to understand how EAB form electrochemically active biofilms (EABFs) on electrodes and how electrons are transferred in EABFs. In addition, since some BESs, e.g. MFCs and MECs for wastewater treatment [41], are operated under continuous flow of electrolytes, it is also important to understand how hydraulic conditions (e.g., static or flow) influence EABF formation. In order to investigate structures, compositions, and electron-transfer mechanisms in live EABFs under electrolyte-flow and non-flow (static) conditions, a study has developed electrochemical flow cells (EFCs), in which MR-1 forms biofilms under electrochemical interaction with WE situated at the bottom of EFC [42]. It has been found that the structure and composition of EABFs formed by MR-1 differ largely depending on growth conditions, including aerobic vs. anaerobic, high vs. low WE potentials, and flow vs. static (Figure 3). For instance, in the presence of a high-potential WE (+0.4 V vs. SHE), MR-1 forms relatively thin (∼10 μm in thickness) EABFs under the flow of electrolytes, in which microbial cells are evenly and densely distributed, and only a small amount of extracellular polysaccharides is present [42]. These features are considered beneficial for MR-1 to exhibit efficient electrochemical interactions with WE. It is however also noteworthy that engineering of strain MR-1 to form thick biofilms is a possible way to facilitate current generation [43,44].

It has also been shown that MR-1 EABF contains flavins that assist in mediated electron transfer between microbial cells and electrodes [34,44]. In addition, a study has found that, in addition to polysaccharides and microbial cells, EABF also contains nucleic acids and proteins, some of which contribute to electron transfer in EABF in some *Shewanella* spp. [45]. Besides, in the initial step of EABF formation by MR-1, pili and extracellular DNA seem to play important roles [46–48], while the *mxdABCD* gene cluster involved in the synthesis of extracellular polysaccharides contributes to the maturation of EABFs [49]. It is also noteworthy that an MR-1 mutant that does not synthesize cell-surface polysaccharides is more adhesive to electrodes than the wildtype and generates more current [50]. A recent work has conducted transcriptomic analyses of EABFs for gaining insights into molecular mechanisms behind EABF formation by MR-1 under electrolyte-flow conditions, suggesting the involvement of several yet-unidentified global regulators, including those categorized into an extracytoplasmic function sigma factor and a diguanylate synthase [51]. A subsequent study has shown that the diguanylate synthase is involved in regulating the global level of cyclic-di-GMP (c-di-GMP) in MR-1 cells and facilitating biofilm formation on electrodes [52]. In addition, another study has also suggested that elevated intracellular c-di-GMP levels in MR-1 increase the expression of c-type cytochromes, thereby correlating the biofilm formation with EET [53]. However, since these studies have been conducted independently using different experimental settings, our knowledge on the formation and functioning of EABFs seems to be fragmentary. Further studies are therefore necessary for gaining comprehensive views on the formation and functioning of EABFs.

## EF and EG, and future perspectives

Although, as described above, substantial amounts of fundamental research have been conducted for understanding molecular mechanisms underlying electrochemical activities of *S. oneidensis* MR-1, attempts to utilize this bacterium for biotechnology are still limited. Since MFCs and MECs have been examined for energy recovery from biomass wastes using naturally occurring microbiomes, and MES has mainly been studied for CO_2_ fixation, MR-1 itself and knowledge obtained from studies on MR-1 are not directly applicable to these processes. On the other hand, several studies have investigated the use of wildtype and engineered MR-1 in EF processes [54–57] that exploit electrodes (either high-potential anode or low-potential cathode) for facilitating oxidative or reductive metabolism (Figure 2). For instance, engineered strains have been examined in anodic EF for producing acetoin from lactate [57] and in cathodic EF for producing isobutanol from a mixture of substrates, including NAG, pyruvate, and lactate under microaerobic conditions [56]. Although these studies are of great value as emerging demonstrations of EF, they were still at the stage of proof of concept, and substantial improvements in production rates are necessary for considering their application to practical processes. For example, the above-mentioned study has reported that the EF process produced 10 mg.l^−1^ isobutanol within 48 h [56], whose production rate is one-hundredth compared with that reported for an isobutanol fermentation process using an engineered yeast [58]. Studies to accelerate production rates in EF are therefore necessary, and these studies will be conducted based on achievements of studies to gain deeper understanding of metabolic and electrochemical characteristics of MR-1.

**Figure 4:**
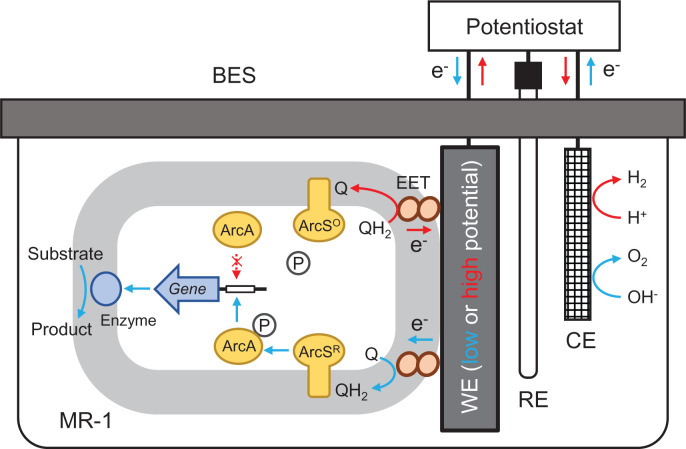
Three-electrode BES designed for EG using the Arc system In the presence of a low-potential electrode, ArcS is reduced (ArcS^R^) by reduced quinones (QH_2_), resulting in the activation of the kinase activity of ArcS and subsequent binding of phosphorylated ArcA to promoter regions of genes under transcriptional control. In contrast, in the presence of a high-potential electrode, ArcS is oxidized (ArcS^O^) by oxidized quinones (Q), resulting in the release of dephosphorylated ArcA from promoter regions. The binding of phosphorylated ArcA may positively or negatively regulate gene expression depending on promoter structures, resulting in up-regulation or down-regulation in the presence of a low-potential electrode.

EG has recently been proposed as a technology to control gene expression using electrodes in BESs [15,59,60], and EG using EAB is based on the finding that the Arc global regulatory system regulates the expression of diverse catabolic genes in MR-1 by sensing electrode potentials [27] (Figure 4). Researchers expect that EG will be used in combination with EF for developing efficient bioproduction processes; e.g., foreign genes that encode bioproduction pathways are expressed under the control of electrode potentials to synchronize gene expression (EG) and energy supply (EF) [15]. Furthermore, Arc-controlled potential-dependent transcription of some catabolic genes can be reprogrammed by EG for developing an efficient bioproduction pathway. These are however still armchair ideas, and we await successful experimental demonstrations of the utility of EG in EB. In addition, given that MR-1 forms different structures of biofilm depending on electrode potentials (Figure 3), it would also be interesting to examine EG for regulating morphogenesis and cell-to-cell communication in EABFs [60].

In a broad sense, EB can also be regarded as a technology for electrochemical control of biological functions (e.g., gene expression, metabolism, morphogenesis, and communication). We consider that this is a new area of research with tremendous future applications that would impact broad areas of biotechnology, including industrial, medical and agricultural biotechnologies. In these studies, *S. oneidensis* MR-1 serves as a bacterial platform that provides researchers with novel interdisciplinary ideas that bridge over electrochemistry and biochemistry.

## Summary

- *S. oneidensis* MR-1 serves as a model electrochemically active bacterium that has extensively been studied for advancing bioelectrochemistry.
- EAB have broad applications, including MFCs, MECs, MES, EF, and EG.
- EB, namely, electrochemical control of biological functions, is a new area of research with tremendous future applications.

## Open Access

Open access for this article was enabled by the participation of University of Tokyo in an all-inclusive *Read & Publish* pilot with Portland Press and the Biochemical Society under a transformative agreement with USACO.

## Abbreviations

Arc: aerobic respiration control
BES: bioelectrochemical system
cAMP/CRP: cyclic AMP/cAMP-receptor protein
CV: cyclic voltammetry
c-di-GMP: cyclic-di-GMP
EAB: electrochemically active bacteria
EABF: electrochemically active biofilm
EB: electro-biotechnology
EET: extracellular electron-transport
EF: electro-fermentation
EFC: electrochemical flow cell
EG: electro-genetics
MEC: microbial electrolysis cell
MES: microbial electrosynthesis
MFC: microbial fuel cell
NAG: N-acetylglucosamine
SHE: standard hydrogen electrode
WE: working electrode
